# Retinal Functional Impairment in Diabetic Retinopathy

**DOI:** 10.3390/biomedicines12010044

**Published:** 2023-12-22

**Authors:** Cornelia Andreea Tănasie, Alexandra Oltea Dan, Oana Maria Ică, Maria Filoftea Mercuț, George Mitroi, Citto-Iulian Taisescu, Veronica Sfredel, Ramona Ingrid Corbeanu, Carmen Luminița Mocanu, Ciprian Danielescu

**Affiliations:** 1 Department of Physiology, University of Medicine and Pharmacy of Craiova, 200349 Craiova, Romania; andreea.tanasie@umfcv.ro (C.A.T.); citto.taisescu@umfcv.ro (C.-I.T.); veronica.sfredel@umfcv.ro (V.S.); ramonna.ingrid@yahoo.com (R.I.C.); 2 Department of Dermatology, University of Medicine and Pharmacy of Craiova, 200349 Craiova, Romania; 3 Department of Ophthalmology, University of Medicine and Pharmacy of Craiova, 200349 Craiova, Romania; maria.mercut@umfcv.ro (M.F.M.); carmen.mocanu@umfcv.ro (C.L.M.); 4 Department of Urology, University of Medicine and Pharmacy of Craiova, 200349 Craiova, Romania; gmitroi@yahoo.com; 5 Department of Ophthalmology, University of Medicine and Pharmacy “Grigore T. Popa”, 700111 Iasi, Romania; ciprian.danielescu@umfiasi.ro

**Keywords:** full-field ERG, diabetes mellitus, diabetic retinopathy

## Abstract

Background: Diabetic retinopathy (DR) is a neurodegenerative disease of the retina. The aim of our study was to analyze latency changes in a full-field electroretinogram (ERG) in patients with type 2 diabetes. Material: This prospective study included 15 diabetic patients without DR, 16 diabetic patients with non-proliferative DR, 14 patients with pre-proliferative DR, 15 patients with proliferative DR, and 14 age-matched controls. All the participants underwent ophthalmologic examination and full-field ERGs. The ERGs were recorded with the Metrovision MonPackOne system. The latencies were analyzed for “a”- and “b”-waves in the dark-adapted (DA) 0.01 ERG, DA 3.0 ERG, DA oscillatory potentials, light-adapted (LA) 3.0 ERG, and 30 Hz flicker ERG. Results: The delayed responses of healthy subjects compared to diabetic patients without DR were the DA oscillatory potentials (25.45 ± 1.04 ms vs. 26.15 ± 0.96 ms, *p* = 0.027). When comparing diabetic patients without DR and with non-proliferative DR, we did not obtain statistically significant delays. Significant delays in the DA 0.01 “b”-wave (61.91 ± 5.52 ms vs. 66.36 ± 8.12 ms, *p* = 0.029), DA 3.0 “b”-wave (41.01 ± 2.50 ms vs. 44.16 ± 3.78 ms, *p* = 0.035), and LA 3.0 “a”-wave (16.21 ± 0.91 ms vs. 16.99 ± 1.16 ms, *p* = 0.045) were found between non-proliferative DR and pre-proliferative DR. When comparing the groups of patients with pre-proliferative DR and proliferative DR, the LA 3.0 ERG “b”-wave (32. 63 ± 2.53 ms vs. 36.19 ± 3.21 ms, *p* < 0.0001), LA 30 Hz flicker ERG “a”-wave (19.56 ± 3.59 vs. 21.75 ± 4.74 ms, *p*= 0.025), and “b”-wave (32.23 ± 4.02 vs. 36.68 ± 3.48 ms, *p* = 0.017) were delayed. Conclusions: the electrophysiological findings from our study indicate that there is a substantial dysfunction of the neural retina in all stages of DR.

## 1. Introduction

Diabetic retinopathy (DR) is one of the most common causes of vision impairment worldwide, affecting one-third of people with diabetes mellitus [1]. The risk of developing DR is correlated with the duration of diabetes and, especially, with blood glucose levels [2]. The risk of blindness in patients with diabetes is 5.2 times higher than in individuals without diabetes [3]. DR affects the retina gradually and can remain unnoticed until the patient experiences severe vision reduction. This is why, once diabetes mellitus is diagnosed, screening for retinal complications is started in order to initiate the correct treatment on time, which consists of maintaining good glycemic control, administering laser photocoagulation, and administering an intravitreal injection with steroids and/or antibodies against a vascular endothelial growth factor [4]. Because it was thought that DR was only a microangiopathy, screening usually consists of morphological investigations, such as fundus photography, optical coherence tomography, and fluorescein angiography, which detect the presence of retinal lesions and aid in disease staging. But some new studies have shown that DR is a chronic neurodegenerative disease of the retina, as DR manifests a significant reduction in the thickness of the inner plexiform layer, inner nuclear layer, and overall retina [5]; increased apoptosis in retinal cells [6]; and glial cell activation [7].

An objective functional examination of the retina is obtained using electroretinography. A full-field electroretinogram is a non-invasive tool for the objective assessment of not only the retinal function but also the individual functionality of photoreceptors, bipolar cells, amacrine cells, and retinal ganglion cells [8,9]. Since clinical changes may not be correlated with the degree of functional retinal damage, the use of functional investigations, such as electroretinography, as a routine investigation, along with the morphological investigations, can provide a better understanding of disease progression. Also, the use of electroretinography could offer effective data for studies that target neural preservation in DR. The aim of our study was to analyze and compare ERG latency changes in patients with diabetes but without DR, patients with non-proliferative DR, patients with pre-proliferative DR, and patients with proliferative DR.

## 2. Materials and Methods

### 2.1. Subjects

A total of 60 type 2 diabetic patients and 14 age-matched controls were enrolled in our study. The patients were divided into 4 subgroups, according to their stage of retinopathy: 15 diabetic patients without DR, 16 diabetic patients with non-proliferative DR, 14 patients with pre-proliferative DR, and 15 patients with proliferative DR, according to the Wilkinson classification [10]. All the diabetic patients had a clinical diagnosis of type 2 diabetes mellitus of at least 10 years.

Subgroup 1 (control) included 28 eyes from 14 healthy subjects. The inclusion criteria were ages between 50 and 80, no diagnosis of diabetes mellitus, no history and no present signs of ophthalmological diseases, and clear optic media. The exclusion criteria were ages lower than 50 and higher than 80, diabetes mellitus, neurological diseases, and any ophthalmological disease.

Subgroup 2 included 30 eyes from 15 patients with type 2 diabetes mellitus without clinical signs of DR (DR-). The inclusion criteria were ages between 50 and 80, a clinical diagnosis of type 2 diabetes mellitus, no history of other ophthalmological diseases, normal findings from ophthalmological investigations, clear optic media, and no signs of DR from an ophthalmoscopic examination. The exclusion criteria were ages lower than 50 and higher than 80, neurological diseases, and ophthalmological diseases.

Subgroup 3 included 32 eyes from 16 patients with type 2 diabetes mellitus with non-proliferative diabetic retinopathy (NDR). The inclusion criteria were ages between 50 and 80; a clinical diagnosis of type 2 diabetes; no history of other ophthalmological diseases; signs of NDR: microaneurysms, retinal hemorrhages, venous loops, exudate or cotton wool spots, and venous beading; and no other ophthalmological diseases. The exclusion criteria were ages lower than 50 and higher than 80, neurological diseases, and ophthalmological diseases, except for NDR.

Subgroup 4 included 28 eyes from 14 patients with type 2 diabetes mellitus with pre-proliferative diabetic retinopathy (PPDR). The inclusion criteria were ages between 50 and 80; a clinical diagnosis of type 2 diabetes; no history of other ophthalmological diseases; clear optic media; signs of PPDR: microaneurysms, retinal hemorrhages, venous loops, exudate or cotton wool spots, venous beading, intraretinal hemorrhages, and intraretinal microvascular abnormalities; and no other ophthalmological diseases. The exclusion criteria were ages lower than 50 and higher than 80, neurological diseases, and ophthalmological diseases, except for PPDR.

Subgroup 5 included 30 eyes from 15 patients with type 2 diabetes mellitus with proliferative diabetic retinopathy (PDR). The inclusion criteria were ages between 50 and 80; clinical diagnosis of type 2 diabetes; no history of other ophthalmological diseases; clear optic media; signs of PDR: microaneurysms, retinal hemorrhages, venous loops, exudate or cotton wool spots, venous beading, intraretinal microvascular abnormalities, and new vessels on disc, new vessels elsewhere, preretinal or vitreous hemorrhages, preretinal fibrosis, and no other ophthalmological disease. The exclusion criteria were ages lower than 50 and higher than 80, neurological diseases, and ophthalmological diseases, except for PDR.

### 2.2. ERG Measurements

All subjects were recruited from the Ophthalmology Outpatient Clinic of Emergency County Hospital of Craiova, Romania, and the “Ocularius” Ophthalmological Research Centre in Craiova, Romania, where the ophthalmological examinations were performed. We recorded the ERGs at Ophthalmological Research Centre “Ocularius”, Craiova, with MonPackOne System (Metrovision, Perenchies, France), according to the ISCEV standards [11]. We used HK loop (“Hawlina-Konec loop”) electrodes as active electrodes and the Ag-AgCl cup type as reference and ground electrodes.

For each participant, the recording protocol consisted of the following:

- Ophthalmological investigation: visual acuity, refraction, anterior segment examination by biomicroscopy, intraocular pressure, fundus examination, and color vision in order to diagnose the stage of diabetic retinopathy and to exclude other ophthalmological diseases.
- ERG recording according to the ISCEV protocol:
  1. Mydriasis using 2.5% phenylephrine eye drops and 1% tropicamide;
  2. Skin preparation for the placement of the electrodes;
  3. Oxybuprocaine chlorhydrate in the lower conjunctival bag;
  4. Electrode settlement: we placed the active electrodes on the lower eyelid bilaterally, the reference electrodes on each orbital rim, and the ground electrode on the vertex;
  5. Twenty minutes of scotopic adaptation;
  6. Scotopic ERG recording: rod response: we used a dim blue flash of 0.01 cd.s.m^−2^, with a 2 s interval between flashes; combined rod–cone response: we used a white flash of 3.0 cd.s.m^−2^, with a 10 s interval between flashes; oscillatory potentials: we used a white flash of 2.0 cd.s.m^−2^, with a 165 s interval between flashes;
  7. Ten minutes of photopic adaptation;
  8. Light-adapted ERG recording: single-flash cone response: we used a 3.0 cd.s.m^−2^ stimulus, with a background luminance of 30 cd.s.m^−2^ and a 0.5 s interval between flashes; 30 Hz flicker: 30 stimuli of 3.0 cd.s.m^−2^ per second.

**Figure 1:**
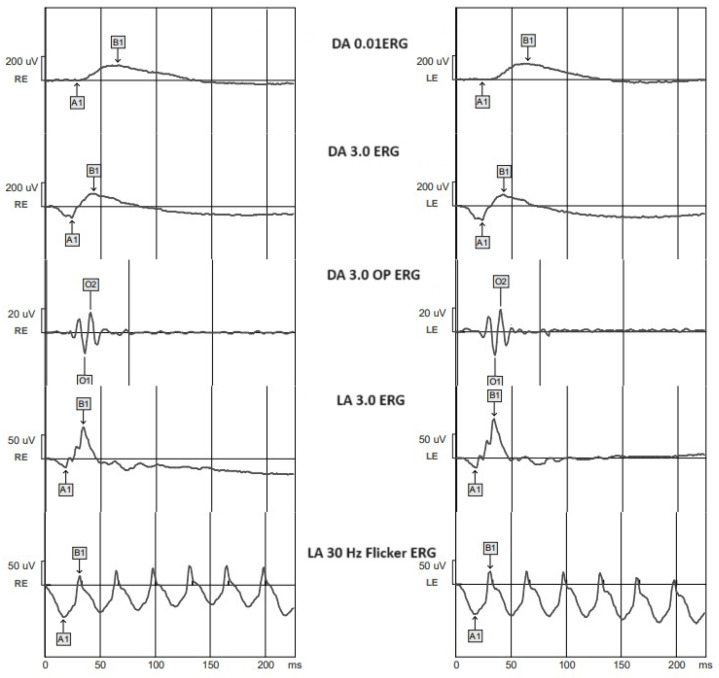
Standard ERG in a healthy individual. DA—dark adapted; LA—light adapted; RE—right eye; LE—left eye; A1—a-wave; B1—b-wave; O1—OP N1; O2—OP N2.

We analyzed the implicit time for a- and b-waves for the DA 0.01, DA 3.0, LA 3.0, and LA 30 Hz flicker ERGs and implicit time for N1 and N2 for DA 3.0 oscillatory potentials (Figure 1). N1 and N2 waves are low-amplitude and high-frequency waves that are superimposed on the rising phase of the b-wave and show the function of the inner retina, especially for the amacrine and horizontal cells [11].

### 2.3. Data Analysis

The obtained data were processed using the Microsoft Excel program (Microsoft Corp., Redmond, WA, USA), with XLSTAT suite for MS Excel (Addinsoft SARL, Paris, France) and the Vision Monitor software Mon2016J integrated in the Metrovision MonPackOne system. The ERG responses were recorded using the MonPackOne program and embedded in the Metrovision MonPackOne acquisition system, which also performed the initial analysis of the signals, the automatic marking, and the manual correction of the landmarks necessary for the quantification of the parameters of the standard electroretinogram waves. All records were stored in the system database, from which they were exported as Excel files for each subject. The secondary processing of the data, the calculation of the fundamental statistical parameters (the mean and the standard deviation of their ratio—the coefficient of variation), and the comparison of the data were carried out by means of ANOVA test, post hoc Fisher LSD test, and Tukey’s test, which is the most used multiple comparison test. Statistical significance was considered at *p* < 0.05.

## 3. Results

For each wave of all standard ERG responses, the mean implicit times and standard deviations were analyzed and compared (Table 1). In our research, we did not obtain statistically significant gender differences, so we analyzed both genders together, for each group.

Due to the small sample size included in our study, we used two multiple comparison tests, Fisher’s post hoc LSD test and Tukey’s test, in order to avoid the errors that could occur and to obtain valid results.

**Figure 2:**
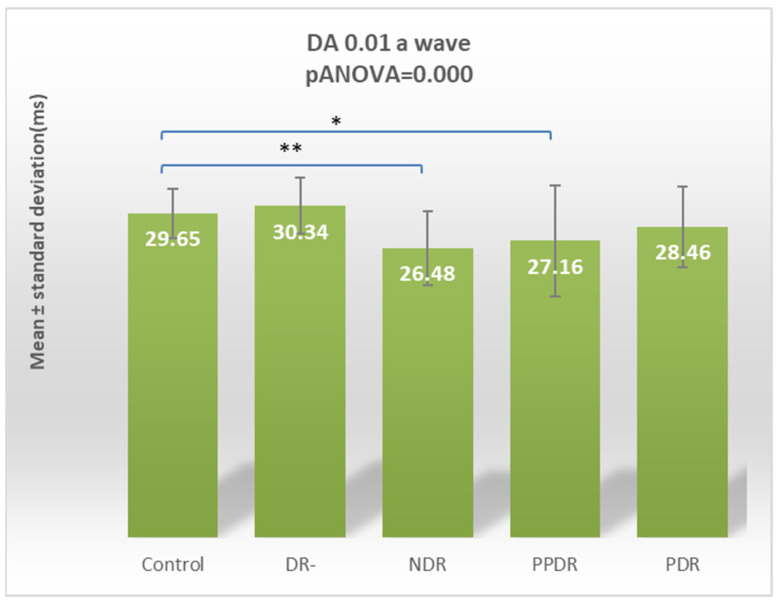
Comparison of mean values of l DA 0.01 a-wave latencies between normal subjects (control) and the subgroups of patients with diabetes mellitus without DR (DR-), patients with non-proliferative diabetic retinopathy (NDR), patients with pre-proliferative diabetic retinopathy (PPDR), and patients with proliferative diabetic retinopathy (PDR). * *p* < 0.05; ** *p* < 0.01.

For the DA 0.01 ERG a-wave, performing the ANOVA test revealed statistically significant differences (*p* ANOVA = 0.000) globally between the five analyzed subgroups. Consequently, Fisher’s LSD post hoc test was used to detect the pairs of subgroups between which these differences were manifested (Table 2). Statistically significant differences were found between the control subgroup and diabetic patients with the NDR (*p* = 0.005) and the control subgroup and patients with PPDR (*p* = 0.022) (Figure 2).

**Figure 3:**
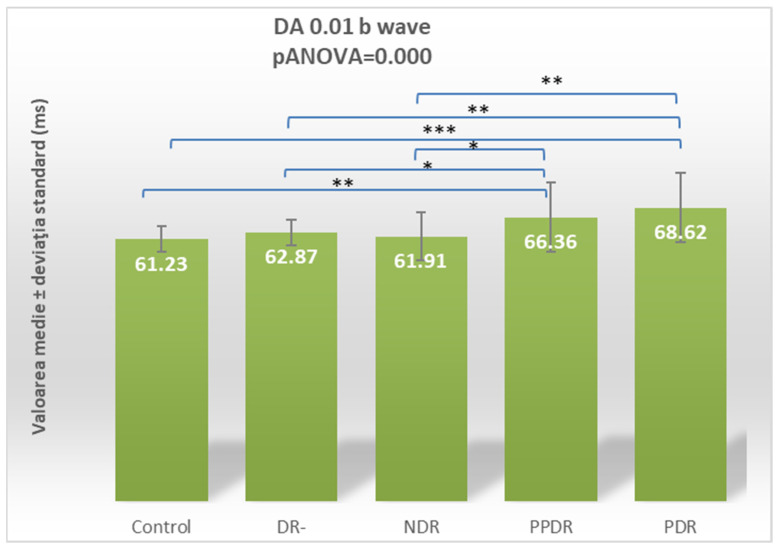
Comparison of mean values of DA 0.01 b-wave latencies between normal subjects (control) and the groups of patients with diabetes mellitus without DR (DR-), patients with non-proliferative diabetic retinopathy (NDR), patients with pre-proliferative diabetic retinopathy (PPDR), and patients with proliferative diabetic retinopathy (PDR). * *p* < 0.05; ** *p* < 0.01; *** *p* < 0.001.

For the DA 0.01 ERG b-wave, the ANOVA test revealed statistically significant differences (*p* ANOVA = 0.000) globally among the five subgroups. Consequently, Fisher’s LSD post hoc test was used to detect the pairs of subgroups between which these differences were manifested (Table 2). Statistically significant differences were found between patients with PPDR and the control (*p* = 0.006), DR- (*p* = 0.0032), and NDR (*p* = 0.029) subgroups and between patients with PDR and the control (*p* < 0.0001), DR- (*p* = 0.001), and NDR (*p* = 0.001) subgroups (Figure 3).

According to Tukey’s test, for the DA 0.01 a-wave, the statistically significant delays are between healthy subjects and patients with NDR (*p* = 0.0405). For the DA 0.01 b-wave, the significant delays are between healthy subjects and PPDR (*p* = 0.0477) and PDR (*p* = 0.0009) groups and between patients with PDR and DR- (*p* = 0.0108) and NDR (*p* = 0.0108) groups (Table 3).

**Figure 4:**
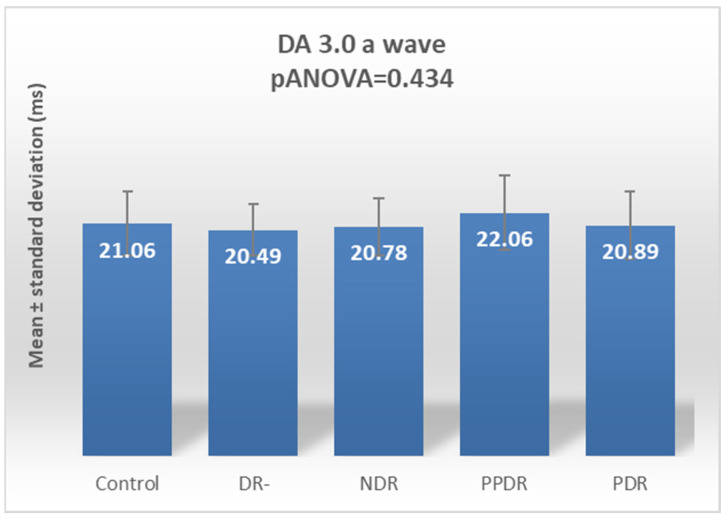
Comparison of mean values of DA 3.0 a-wave latencies between healthy subjects (control) and the subgroups of patients with diabetes mellitus without DR (DR-), patients with non-proliferative diabetic retinopathy (NDR), patients with pre-proliferative diabetic retinopathy (PPDR), and patients with proliferative diabetic retinopathy (PDR).

For the DA 3.0 ERG a-wave, the ANOVA test revealed no statistically significant differences globally between the five analyzed subgroups (*p* = 0.434) (Table 2, Figure 4).

**Figure 5:**
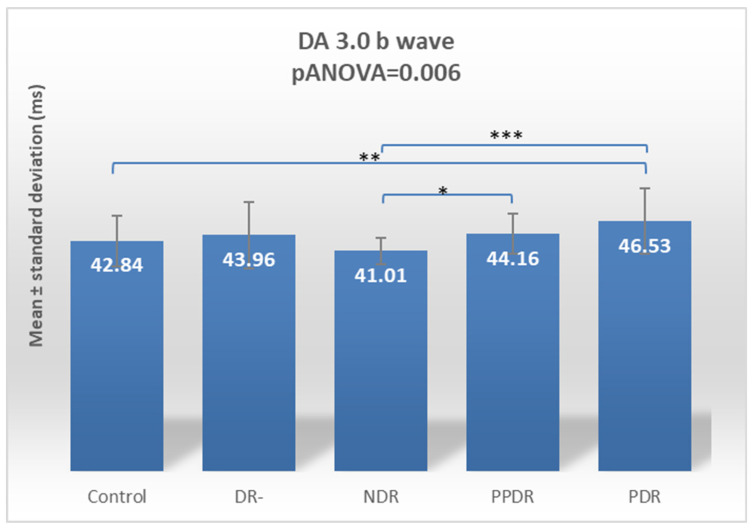
Comparison of mean values of DA 3.0 b-wave latencies between healthy subjects (control) and the groups of patients with diabetes mellitus without DR (DR-), patients with non-proliferative diabetic retinopathy (NDR), patients with pre-proliferative diabetic retinopathy (PPDR), and patients with proliferative diabetic retinopathy (PDR). * *p* < 0.05; ** *p* < 0.01; *** *p* < 0.001.

The ANOVA test for the DA 3.0 b-wave revealed statistically significant differences (*p* ANOVA = 0.006) globally between the five analyzed subgroups. By using the Fisher LSD post hoc test, statistically significant differences were found between patients with PDR and subjects from the control (*p* = 0.009) and NDR (*p* = 0.0003) subgroups and between patients with NDR and patients with PPDR (*p* = 0.035) (Table 2) (Figure 5).

According to Tukey’s test, for the DA 3.0 a-wave, there is no statistically significant delay between groups. For the DA 3 b-wave, the significant delay is between the NDR and PDR groups (*p* = 0.0025) (Table 3).

**Figure 6:**
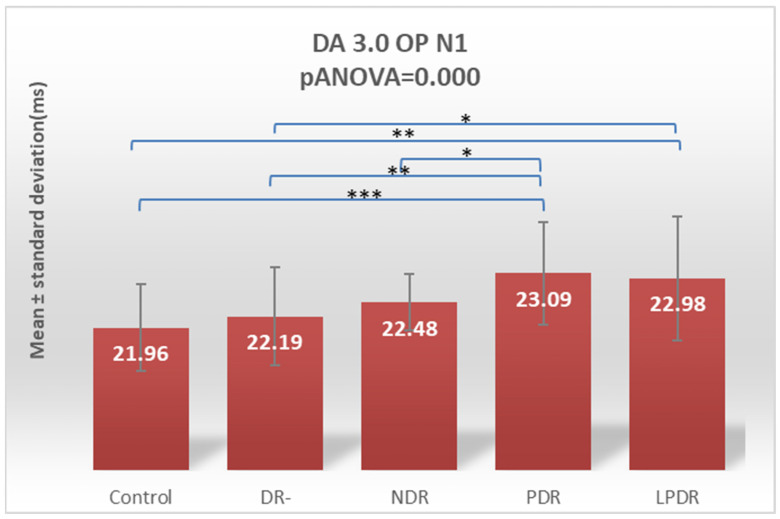
Comparison of mean values of DA 3.0 OP N1 wave latencies between healthy subjects (control) and the groups of patients with diabetes mellitus without DR (DR-), patients with non-proliferative diabetic retinopathy (NDR), patients with pre-proliferative diabetic retinopathy (PPDR), and patients with proliferative diabetic retinopathy (PDR). * *p* < 0.05; ** *p* < 0.01; *** *p* < 0.001.

**Figure 7:**
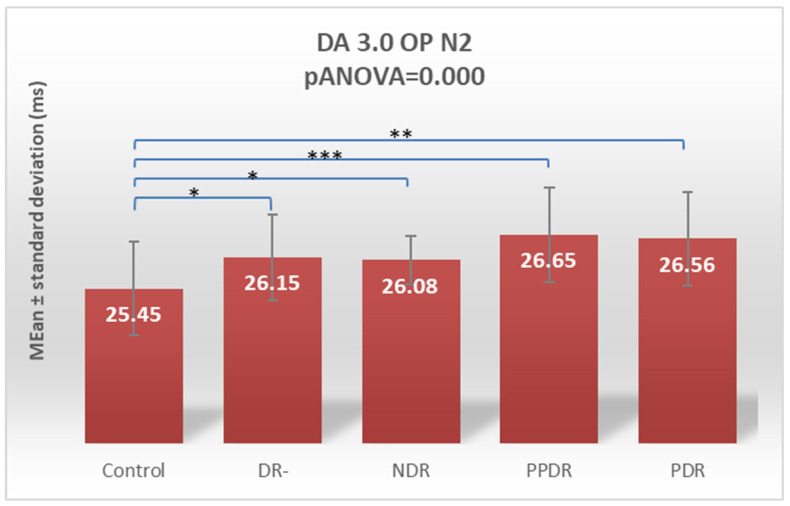
Comparison of mean values of DA 3.0 OP N2 wave latencies between healthy subjects (control) and the groups of patients with diabetes mellitus without DR (DR-), patients with non-proliferative diabetic retinopathy (NDR), patients with pre-proliferative diabetic retinopathy (PPDR), and patients with proliferative diabetic retinopathy (PDR). * *p* < 0.05; ** *p* < 0.01; *** *p* < 0.001.

The oscillatory potentials (OPs) are characterized by a highly significant overall increase in latency for both the N1 and N2 waves, with ANOVA *p* values of 0.000 (Figure 6 and Figure 7). The analysis of the five subgroups, using the Fisher LSD post hoc test, showed that for the N1 wave, significant differences were found between the PPDR and the control subgroups (*p* = 0.0001) and the DR- (*p* = 0.002) and NDR (*p* = 0.043) subgroups, as well as between the PDR and the control (*p* = 0.002) subgroups and DR- (*p* = 0.016) subgroups (Table 2).

For the N2 wave, the Fisher LSD post hoc test showed significant changes between the control subgroup and all four other groups: DR- (*p* = 0.027), NDR (*p* = 0.045), PPDR (*p* = 0.0001), and PDR (*p* = 0.001) (Table 2).

Performing Tukey’s test for both N1 and N2 waves of DA 3OP, the statistically significantly increased latencies are between healthy subjects and PPDR (*p* = 0.009 and 0.008, respectively) and PDR (*p* = 0.0145 and 0.0096, respectively) groups and between DR- and PPDR groups (*p* = 0.0195 and 0.0179, respectively) (Table 3).

**Figure 8:**
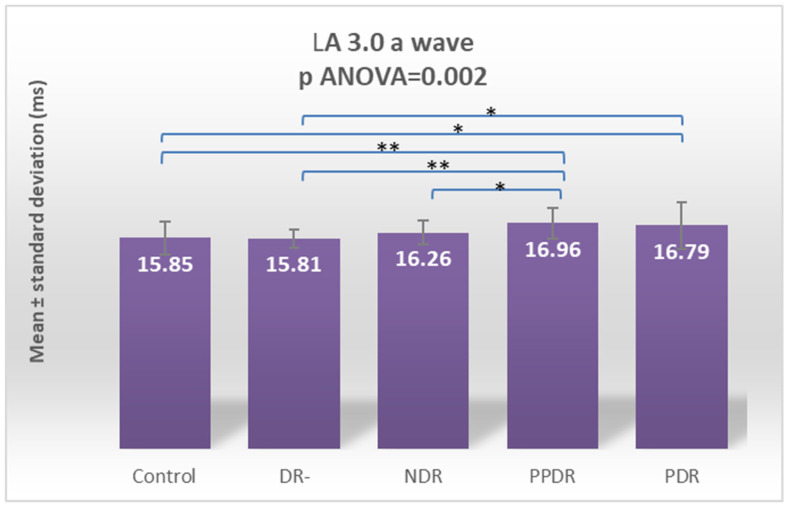
Comparison of mean values of LA 3.0 a-wave latencies between healthy subjects (control) and the groups of patients with diabetes mellitus without DR (DR-), patients with non-proliferative diabetic retinopathy (NDR), patients with pre-proliferative diabetic retinopathy (PPDR), and patients with proliferative diabetic retinopathy (PDR). * *p* < 0.05; ** *p* < 0.01.

**Figure 9:**
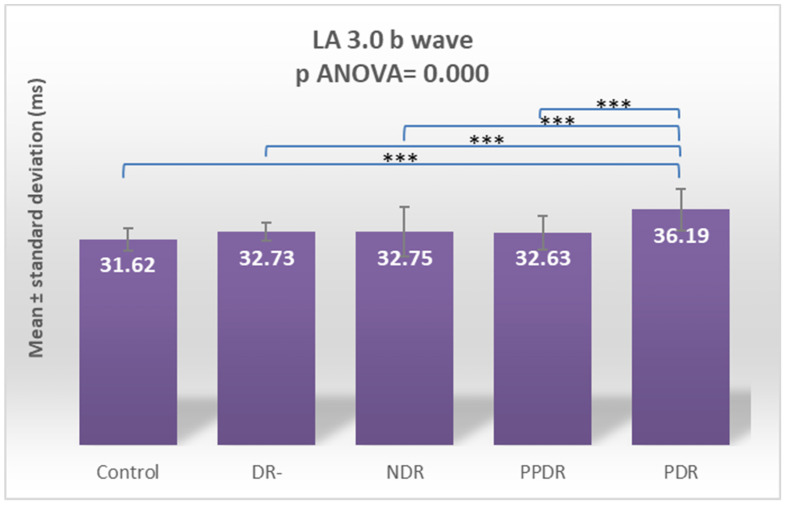
Comparison of mean values of LA 3.0 b-wave latencies between healthy subjects (control) and the groups of patients with diabetes mellitus without DR (DR-), patients with non-proliferative diabetic retinopathy (NDR), patients with pre-proliferative diabetic retinopathy (PPDR), and patients with proliferative diabetic retinopathy (PDR). *** *p* < 0.001.

Analyzing the a- and b-waves in the standard LA 3.0 ERG recording, a significant change in latency for both the a-wave (*p* ANOVA = 0.002) and b-wave (*p* ANOVA = 0.000) were found (Figure 8 and Figure 9). For the a-wave, this overall difference was due to significant increases in latency between the subgroup of subjects with PPDR versus healthy subjects (*p* = 0.002) and the subgroup with type 2 diabetes mellitus but without diabetic retinopathy (*p* = 0.002) and subjects with PDR versus subjects in the control (*p* = 0.009) and DR- (*p* = 0.009) subgroups (Figure 8). For the b-wave, the difference is due to the major increase in latency in the subgroup of subjects with PDR compared to the other four groups included in the study: control (*p* < 0.0001), DR- (*p* < 0.0001), NDR (*p* < 0.0001), and PPDR (*p* < 0.0001) (Table 2) (Figure 9).

Performing Tukey’s test for the LA 3 a-wave, the significant delays are between PPDR patients and the control (*p* = 0.0130) and DR- (*p* = 0.0143) subjects. For the LA 3 b-wave, the delays are between PDR and all the other four groups: control (*p* < 0.0001), DR- (*p* = 0.012), NDR (*p* = 0.0015), and PPDR (*p* = 0.0010) (Table 3).

**Figure 10:**
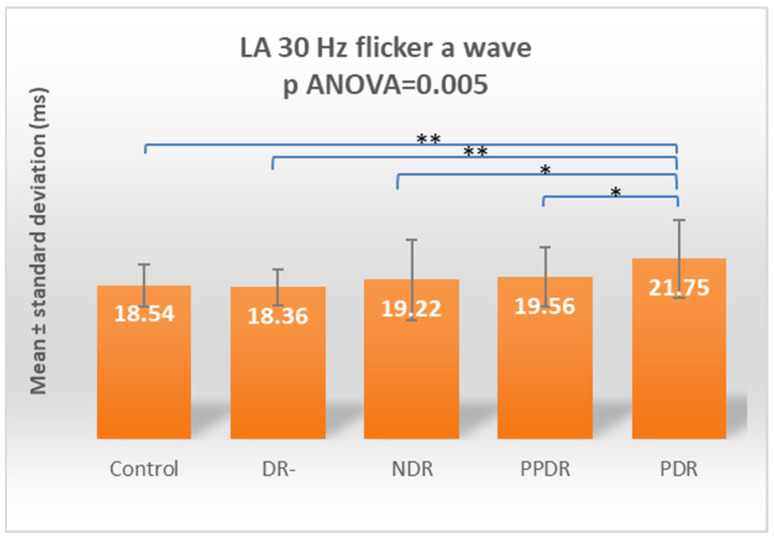
Comparison of mean values of LA 30 Hz flicker a-wave latencies between healthy subjects (control) and the groups of patients with diabetes mellitus without DR (DR-), patients with non-proliferative diabetic retinopathy (NDR), patients with pre-proliferative diabetic retinopathy (PPDR), and patients with proliferative diabetic retinopathy (PDR). * *p* < 0.05; ** *p* < 0.01.

By analyzing the a-wave from the LA 30 Hz flicker ERG, we found a globally significant difference between the five study subgroups (*p* ANOVA = 0.005). The Fisher LSD post hoc test showed that this overall difference was due to increased latency in the subgroup of subjects with PDR compared to the control (*p* = 0.004), DR- (*p* = 0.002), NDR (*p* = 0.013), and PPDR (*p* = 0.025) (Table 2) (Figure 10).

**Figure 11:**
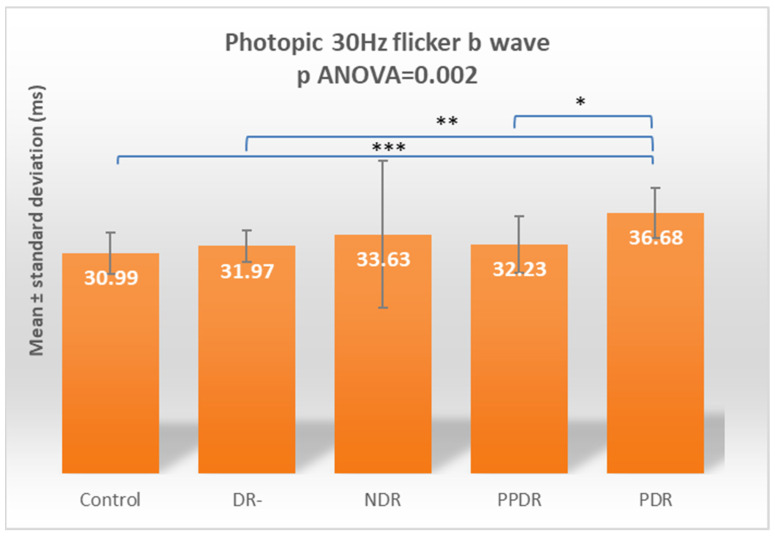
Comparison of mean values of LA 30 Hz flicker b-wave latencies between healthy subjects (control) and the groups of patients with diabetes mellitus without DR (DR-), patients with non-proliferative diabetic retinopathy (NDR), patients with pre-proliferative diabetic retinopathy (PPDR), and patients with proliferative diabetic retinopathy (PDR). * *p* < 0.05; ** *p* < 0.01; *** *p* < 0.001.

For the b-wave, the globally significant difference (*p* ANOVA = 0.002) was due to the increased implicit time between subjects from the PDR subgroup and those in the control (*p* = 0.0005), DR- (*p* = 0.005), and PPDR (*p* = 0.017) subgroups (Table 2) (Figure 11).

According to Tukey’s test, for 30 Hz flicker, both a- and b-waves have statistically significant delays between PDR and control (*p* = 0.0347 and 0.0041, respectively) and DR- (*p* = 0.0155 and 0.0371, respectively) groups (Table 3).

## 4. Discussion

The objectives of this study were to observe the electroretinographic evolution of DR and to obtain a comparison between the various stages of DR regarding retinal functional aspects, while excluding changes due to age, by comparing the diabetic patients with a subgroup of subjects similar in age.

All parameters analyzed in our study showed a statistically significant global delay, except for the a-wave from the Scotopic 3.0 recording of the standard electroretinogram, which assesses the mixed rod–cone response.

The first electroretinographic change identified is the increase in the implicit time of the second wave of OP in patients with type 2 diabetes without diabetic retinopathy compared to the healthy subjects. In our study between healthy subjects and those with diabetes without diabetic retinopathy, we did not find other statistically significant changes.

The results of our research correlate with the majority of studies that claim that OPs represent the most sensitive electroretinographic indicator in diabetic eye disease [12,13,14,15,16], demonstrating an early impairment of neuronal synaptic activity of amacrine and horizontal retinal cells.

Yonemura was the first to show that OPs are impaired in more than 50% of patients with diabetes without DR [17]. Holopigian showed that the latency of OP was not significantly altered in diabetics compared to healthy subjects; only the amplitude was decreased, indicating that the OPs are the first parameter impaired in the ERG [18]. In a study on rats, in which diabetes was induced by an injection of streptozotocin, Hancock demonstrated a relationship between OP and the appearance and evolution of DR lesions [19]. More recently, Gualtieri also showed OP abnormalities in 82.95% of subjects with diabetes mellitus without DR [20].

In subjects with NDR, we did not obtain statistically significant delays, but we observed a slight increase in the a-wave of the rod–cone response and a-wave of the cone response. As is known, the scotopic a-wave reflects the activity of the photoreceptors, in our case both rods and cones, but also has postreceptoral contributions [21]. Hanock showed a delay in DA 3.0 a-wave in diabetic patients without DR in his study [19]. In our research, this change appeared later in DR evolution in patients with NDR, showing a slightly decreased activity of rods [4,21]. We also obtained a discrete delay of the response of cone cells. Usually, cone damage occurs in the more advanced stages of diabetic retinopathy; however, Gualtieri et al. have found evidence of damage to cone cells in the early stages [20]. These small differences in ERG parameters between DR- and NDR could be due to the small sample size included in our study but also due to variability in patient populations.

Our results are consistent with other studies that found dark-adapted ERG delays in the early stages of DR [22,23]. The inferred pathway for this ERG change is the increased metabolic demand and high oxidative stress of the diabetic retina under scotopic conditions [24,25].

In the group of subjects with PPDR, the main increases in latency were the b-wave of the rod cell response and the mixed rod–cone response and the “a”-wave of the cone cell response. Thus, as the ischemia progressed, we observed a marked alteration of the scotopic system with damage to the internal retinal layers but also damage to the photoreceptors in the photopic system.

Comparing our results with those of the study published by Luu et al., which included 18 patients with diabetes without DR and 10 patients with NDR, both studies show that the photopic system is not affected in the early stages of DR. On the other hand, Luu’s study showed the impairment of the b-wave in the scotopic system before the onset of DR and NDR; in our research, the b-wave shows a significant increase in latency, with the appearance of a pre-proliferative stage [24]. Both studies have a small number of participants and the same ERG recording protocol, so this difference could be due to patients’ variability and statistical methods.

Overall, with the progress of ischemia, our research showed minimal cone cell damage, in addition to marked alteration of the scotopic system. This fact is in agreement with other studies that reported cone pathway abnormalities in subjects with advanced DR [26,27,28].

In the group of subjects with PDR, the affected waves were the LA 3.0 b-wave and LA 30 Hz flicker a- and b-waves, showing a severe impairment of the photopic system.

The flicker stimulation assesses neurovascular coupling, that is, the ability to adjust blood flow in the microcirculation of the neuronal retina in response to neuronal activity. The pathway involved in flicker stimulation changes is that an increased frequency of the light stimulus induces greater activity in nervous cells, with increased release of nitric oxide and other vasodilatory substances, increasing the microvascular response [29,30].

In fact, two mechanisms contribute to the basis of this altered response: firstly, the alteration of the function of photoreceptors and retinal neurons and, secondly, the impairment of the retinal microcirculation, creating a vicious circle [31,32,33].

In our research, the flicker response was delayed in subjects with PDR, a fact that is in agreement with other studies that report an increased implicit time in diabetics with advanced stages of DR [34,35,36,37,38] and are normal or minimally affected in early stages [39,40,41,42,43].

By using Tukey’s test, we obtained only a significant delay of the scotopic a-wave for patients with NDR compared to healthy subjects, also showing a slight impairment of rods in early stages of DR. With the disease progression to a pre-proliferative stage, we also obtained a delay of the b-wave within the rod pathway and the a-wave within the cone system. We obtained a different result regarding oscillatory potentials—these being delayed in patients with PPDR. For the PDR group, we also obtained a severe impairment of cone pathway, through a significant delay of the LA 3 a-wave and LA 30 Hz flicker a- and b-waves.

Research over the past 20 years has shown that normal or slightly delayed a- and b-waves are consistently presented during the early stages of DR. However, studies examining more advanced DR stages or combinations of subjects across multiple DR stages have reported delayed b-waves. There is a lack of large-scale clinical studies of electroretinography conducted in diabetic patients able to clearly define the disease stages. Further research is necessary to clarify questions regarding the extent to which the a- and b-waves are abnormal in diabetic individuals who have different disease severity.

The correspondence in clinical practice of the ERG for diabetic patients is related to quantifying neural dysfunction prior to the appearance of the first signs of retinopathy, which would help set individual goals for better glycemic control. Further research is necessary to determine whether diabetic patients who have ERG abnormalities are more likely to progress in a more advanced stage and therefore adjust the optimal individualized follow-up intervals for diabetic patients without clinically apparent retinopathy. Also, patients with flicker ERG abnormalities may show a risk of developing pre-proliferative or proliferative DR. This marker could indicate the need to decrease the duration between the usual check-ups and initiate laser therapy as soon as the neovascularization appears. Thus, electroretinography can strengthen the relationship between the ophthalmologist and the diabetologist in the sense of individualized therapy in correlation not only with glycemic values but also with preclinical retinal complications.

Considering that electroretinography is a technique not very often used in diabetic ophthalmological pathology and that most studies [3,44] evaluated electroretinographic changes, especially in the early stages, we can say that this research is one of the few studies to have followed the evolution of retinal function impairment in one of the most common complications of diabetes. Another important feature of our study is the highlighting of the different effects on rod cells versus cone cells as the disease progresses.

Based on the ERG changes obtained in our research, as the DR progresses, the following elements are affected: amacrine and horizontal cells, photoreceptors from the scotopic system followed by the inner retinal layers of the rods pathway, photoreceptors from the photopic system, and ON and OFF bipolar cells within the cone pathway. The early ERG sign that we have signaled in patients with type 2 diabetes without DR is the increase in the implicit time of the second wave of OP. In subjects with NDR, we did not obtain statistically significant delays, but we observed a slight increase in the “a”-wave of the rod–cone response and the “a”-wave of the cone response. There was a significant increase in the latency of the dark-adapted 0.01 “b”-wave and 3.0 “b”-wave from the early to the late stages of DR. In the group of subjects with PDR, the affected waves were the LA 3.0 b-wave and LA 30 Hz flicker “a”- and “b”-waves, showing a severe impairment of the photopic system.

A limitation of the current study could be the small sample size for each group: 14 participants for the DR- group, 15 for the NDR group, and 16 and 15 patients for the PPDR and PDR groups, respectively. It is well known that studies with a small sample size may not be able to detect a proper difference between groups due to inaccurate statistics leading to erroneous conclusions [45]. Large sample size studies could offer more accurate data regarding inferential statistics. We tried to neutralize the errors that could appear due to small sample size by using two types of multiple comparison tests, and we obtained similar results. Also, we analyzed a parameter, the implicit time of all standard ERG waves, which is stable compared to the amplitude of the waves, which has a higher variability. It is considered that studies with a small sample size are required to evaluate stable parameters [45]. Even if the small number of subjects could be a limitation of our study, due to the fact that we investigated a stable parameter and we used two multiple comparison tests, we consider our results and conclusions accurate.

Another limitation is that the participants were not monitored over a longer period of time through electroretinography so that we could evaluate the retinal progression of the disease. Although this investigation is non-invasive, the patients show some reluctance to repeat this investigation due to the increased examination time, approximately one hour, as well as slight eye discomfort due to the induced mydriasis and to the introduction of the electrodes into the conjunctival sac.

## 5. Conclusions

The standard ERG is an objective test able to assess global retinal dysfunction, giving us important insights into the neural effects of diabetes mellitus on the retinal cells that may not be similar to the clinically visible lesions. Our study showed that, as DR progresses, the following elements are affected: amacrine and horizontal cells and the rod pathway followed by the cone pathway. The obtained results support the idea that electroretinography is a useful technique that can be used not only in research but also in current practice and could help to set individual goals for better control of the disease.

## Figures and Tables

**Table 1:** Mean implicit times and standard deviations for healthy subjects (control), patients with diabetes mellitus without DR (DR-), patients with diabetes mellitus and NDR (NDR), patients with diabetes mellitus and PPDR (PPDR), and patients with diabetes mellitus and PDR (PDR). a—a-wave; b—b-wave.

Parameter	CONTROL	DR-	NDR	PPDR	PDR
DA 0.01 ERG a	29.65 ± 2.29	30.34 ± 2.57	26.48 ± 3.36	27.16 ± 5.06	28.46 ± 3.70
DA 0.01 ERG b	61.23 ± 2.99	62.87 ± 2.99	61.91 ± 5.52	66.36 ± 8.12	68.62 ± 8.01
DA 3.0 ERG a	21.06 ± 2.92	20.49 ± 2.30	20.78 ± 2.60	22.06 ± 3.35	20.89 ± 3.04
DA 3.0 ERG b	42.84 ± 4.80	43.96 ± 6.19	41.01 ± 2.50	44.16 ± 3.78	46.53 ± 6.11
OP N1	21.96 ± 0.89	22.19 ± 1.02	22.48 ± 0.58	23.09 ± 1.06	22.98 ± 1.28
OP N2	25.45 ± 1.04	26.15 ± 0.96	26.08 ± 0.54	26.65 ± 1.05	26.56 ± 1.04
LA 3.0 ERG a	15.85 ± 1.26	15.81 ± 0.69	16.21 ± 0.91	16.99 ± 1.16	16.79 ±1.73
LA 3.0 ERG b	31.62 ± 1.75	32.73 ± 1.40	32.75 ± 3.76	32.63 ±2.53	36.19 ± 3.21
LA 30 Hz Flicker a	18.54 ± 2.50	18.36 ± 2.16	19.22 ± 4.85	19.56 ± 3.59	21.75 ± 4.74
LA 30 HZ Flicker b	30.99 ± 2.87	31.97 ± 2.28	33.63 ± 10.37	32.23 ± 4.02	36.68 ± 3.48

**Table 2:** Fisher LSD post hoc test *p* values for the DA 0.01 a- and b-waves, DA 3.0 a- and b-waves, OP N1 and N2 waves, LA 3 a- and b-waves, LA 30 Hz flicker a- and b-waves for the comparisons between normal subjects (control), patients with diabetes mellitus without DR (DR-), patients with diabetes mellitus and NDR (NDR), patients with diabetes mellitus and PPDR (PPDR), and patients with diabetes mellitus and PDR (PDR). a—a-wave; b—b-wave.

Contrast	DA 0.01 a	DA 0.01 b	DA 3.0 a	DA 3.0 b	DA 3.0 OP N1	DA 3.0 OP N2	LA 3.0 a	LA 3.0 b	LA 30 a	LA 30 b
Control vs. NDR	0.005	0.663	0.746	0.183	0.064	0.045	0.272	0.126	0.756	0.076
Control vs. PPDR	0.022	0.006	0.231	0.357	0.0001	0.0001	0.002	0.163	0.557	0.269
Control vs. DR-	0.214	0.592	0.442	0.431	0.400	0.027	0.877	0.146	0.542	0.681
Control vs. PDR	0.255	<0.0001	0.831	0.009	0.002	0.001	0.009	<0.0001	0.004	0.0005
PDR vs. NDR	0.094	0.001	0.916	0.0003	0.143	0.152	0.142	<0.0001	0.013	0.074
PDR vs. PPDR	0.241	0.334	0.183	0.114	0.674	0.770	0.582	<0.0001	0.025	0.017
PDR vs. DR-	0.922	0.001	0.599	0.083	0.016	0.160	0.009	<0.0001	0.002	0.005
DR- vs. NDR	0.114	0.935	0.674	0.046	0.312	0.753	0.237	0.941	0.392	0.226
DR- vs. PPDR	0.279	0.0032	0.066	0.891	0.002	0.065	0.002	0.954	0.270	0.547
PPDR vs. NDR	0.629	0.029	0.152	0.035	0.043	0.053	0.045	0.895	0.793	0.519

**Table 3:** Tukey’s test *p* values for the DA 0.01 a- and b-waves, DA 0.01 a- and b-waves, OP N1 and N2 waves, LA 3 a- and b-waves, and LA 30 Hz flicker a- and b-waves for the comparisons between normal subjects (control), patients with diabetes mellitus without DR (DR-), patients with diabetes mellitus and NDR (NDR), patients with diabetes mellitus and PPDR (PPDR), and patients with diabetes mellitus and PDR (PDR). a—a-wave; b—b-wave.

Contrast	DA 0.01 a	DA 0.01 b	DA 3.0 a	DA 3.0 b	DA 3.0 OP N1	DA 3.0 OP N2	LA 3.0 a	LA 3.0 b	LA 30 a	LA 30 b
Control vs. DR-	0.7223	0.9832	0.9386	0.9329	0.9151	0.9127	0.9999	0.1017	0.9728	0.9938
Control vs. NDR	0.0405	0.9923	0.9976	0.6663	0.3338	0.2596	0.8042	0.0858	0.9979	0.3778
Control vs. PPDR	0.1463	0.0477	0.7491	0.8868	0.0009	0.0008	0.0130	0.1155	0.9762	0.7986
Control vs. PDR	0.7820	0.0009	0.9995	0.0700	0.0145	0.0096	0.0654	<0.0001	0.0347	0.0041
DR- vs. NDR	0.5034	>0.9999	0.9933	0.2663	0.8468	0.7777	0.7573	>0.9999	0.9099	0.7380
DR- vs. PPDR	0.8128	0.1977	0.3459	>0.9999	0.0195	0.0179	0.0143	>0.9999	0.7999	0.9738
DR- vs. PDR	>0.9999	0.0108	0.9845	0.4089	0.1322	0.0956	0.0655	0.0012	0.0155	0.0371
NDR vs. PPDR	0.9886	0.1864	0.6018	0.2161	0.2475	0.2963	0.2592	>0.9999	0.9989	0.9663
NDR vs. PDR	0.4451	0.0108	>0.9999	0.0025	0.6388	0.6147	0.5790	0.0015	0.1029	0.3832
PPDR vs. PDR	0.7625	0.8677	0.6680	0.5061	0.9812	0.9952	0.9816	0.0010	0.1778	0.1185

## Data Availability

The authors declare that the data for this research are available from the corresponding authors upon reasonable request.
